# Planning Adjustment of Toric Capsular Bag Intraocular Lens Axis to Minimise Refractive Cylinder Outcome—A Calculation Concept Based on Vergence Transformations

**DOI:** 10.3390/diagnostics16071029

**Published:** 2026-03-30

**Authors:** Achim Langenbucher, Nóra Szentmáry, Alan Cayless, Giacomo Savini, Iwan Bolzern, Benjamin Fassbind, Peter Hoffmann, Jascha Armin Wendelstein

**Affiliations:** 1 Department of Experimental Ophthalmology, Saarland University, 66424 Homburg (Saar), Germany; nszentmary@gmail.com (N.S.); iwan.bolzern@gmail.com (I.B.); benjamin.fassbind@hotmail.com (B.F.); wendelsteinjascha@gmail.com (J.A.W.); 2 Department of Ophthalmology, Semmelweis-University, 1085 Budapest, Hungary; 3 School of Physical Sciences, The Open University, Milton Keynes MK7 6AA, UK; alan.cayless@gmail.com; 4 IRCCS Bietti Foundation, 00198 Rome, Italy; giacomo.savini@startmail.com; 5 Augen- und Laserklinik Castrop-Rauxel, 44575 Castrop-Rauxel, Germany; pupillenpeter@gmx.de; 6 Department of Ophthalmology, Ludwig-Maximilian University, 80336 Munich, Germany

**Keywords:** toric intraocular lenses, toric IOL axis adjustment, spectacle refraction, paraxial optics, vergence calculation, ELP prediction model

## Abstract

**Purpose:** The aim of this study was to develop a concept for adjustment planning of intraocular lens orientation axes after cataract surgery with implantation of toric intraocular lenses (tIOLs) and to predict the spectacle refraction after tIOL re-alignment. **Methods:** This calculation concept based on paraxial spherocylindrical vergence transformations uses the actual spherocylindrical refraction at the spectacle plane, corneal power, and the labelled power and measured axis of the implanted tIOL to minimise the refractive cylinder by simulating the rotation of the tIOL in the eye. The axial lens position is derived from simple prediction models using anterior chamber depth and lens thickness or axial length from preoperative biometry or the equivalent tIOL power. The new target axis is predicted together with the spherocylindrical refraction after re-alignment of the tIOL. **Results:** To show the applicability of this calculation model, we provide four clinical working examples: example 1 deals with keratometric power values; example 2 deals with keratometric curvature values, including surgically induced astigmatism and a statistical posterior astigmatism correction for the cornea (both examples with a thin cornea model); example 3 deals with corneal curvature data for the front and back surface; and example 4 deals with keratometric power data and corneal back surface power data, including surgically induced astigmatism (both examples with a thick cornea model). **Conclusions:** The effect of tIOL axis adjustment after cataract surgery can be predicted based on actual refraction, corneal power, tIOL power and the measured axis, and a simulation of the tIOL axis rotation enables the best orientation with the lowest refractive cylinder at the spectacle plane to be found.

## 1. Introduction

Toric intraocular lenses are gaining popularity in clinical routine. Today, 5–10% of intraocular lenses (IOLs) used in cataract surgery include some amount of toric correction. This is especially the case in the premium lens segment, where implants providing pseudophakic accommodation, such as lenses with elongated focus or multifocal lenses, are often prescribed. In these types of lenses, indications for a toric correction begin with corneal astigmatism values as low as 0.75 dioptres (D) [1,2].

However, in contrast to the implantation of rotationally symmetric lenses, implantation of toric IOLs (tIOLs) is more challenging, and the lens power calculation during surgical planning is more critical, as is the implantation itself. Hence, only around 55–65% of tIOL implantations achieve a postoperative astigmatism magnitude below 0.5 D. As a minimum, tIOL calculation requires keratometric curvature or refractive power data from both corneal meridians, including axis orientation. Statistical correction strategies for the corneal back surface astigmatism or direct consideration of corneal back surface curvature or power from a tomographer, together with an optional consideration of the surgically induced astigmatism as vectorial superposition, could improve the refractive outcome [3,4,5].

During surgery, the surgeon aligns the flat axis of the tIOL, as marked by the manufacturer, to the steep axis of the cornea [1,2,5], but even with perfect alignment of the tIOL axis, we sometimes observe some rotation of the tIOL in the capsular bag away from the target axis [1,2,6,7,8,9]. Such a misalignment of the tIOL axis could happen in the short term (e.g., in the first hour after surgery) or in the long term, either days or weeks after tIOL implantation. A misalignment of the tIOL axis does not normally affect the spherical equivalent refraction at the spectacle plane, but it can result in a mixed cylinder in the spectacle refraction from the corneal astigmatism (acting at corneal plane) and the crossed toric correction of the tIOL at the lens plane. Such a cylindrical telescope setup could also induce anamorphic distortions, and for larger axis deviations of the tIOL, a re-alignment of the tIOL axis is indicated, especially with larger toric corrections [3,4].

In clinical practice there are no strict benchmarks as to the amount of axis rotation or toric correction that would indicate re-alignment of the tIOL [10,11,12]. However, the large amount of residual astigmatism after tIOL implantation suggests that clinicians require planning tools for this eventuality. Such tools would ideally be based on the already existing biometric measurements performed for lens power calculation and would be able to indicate how much the tIOL should be rotated clockwise or counter-clockwise, together with a prediction of the spectacle refraction after re-alignment of the tIOL [12,13,14]. As a further benefit, any clinical tool should be able to predict how much improvement the patient could expect in terms of reduction in the refractive cylinder at the spectacle plane.

From optics, it is clear that the amount of astigmatic correction of a tIOL considered at the corneal plane depends on the ray vergence entering the cornea, the corneal refraction, and the distance between the cornea and the tIOL [15,16]. The ray vergence entering the cornea is determined by the object vergence (e.g., 0 for objects at infinity), the spherocylindrical spectacle correction, and the vertex distance [3,4]. In the simplest case, the cornea could be interpreted in terms of its keratometric measures as derived from the front surface data (with or without statistical back surface astigmatism correction), but we could also consider the cornea as a thick lens with front and back surface measures [15,17]. And finally, we should use a realistic model for the distance between the cornea and the lens (the effective lens position, ELP, mostly interpreted as the distance from the corneal front apex to the image-sided principal plane of the IOL). This ELP could, for example, be calculated using a modern lens power formula [18,19,20,21,22].

The purpose of the present study was to develop a concept to predict the following:

- The amount of rotation and the target axis of a toric lens implant which has to be re-aligned after cataract surgery for a potential misalignment in the toric axis.
- The spectacle refraction after the re-alignment procedure and the potential gain in performance in terms of a relative reduction in the refractive cylinder.

We predict these factors based on the preoperative biometry, the power and measured axis of the toric lens, and the measured spectacle refraction. We also present four clinical examples demonstrating the applicability of the calculation concept.

## 2. Materials and Methods

The calculation strategy in this simulation study is based on

- The biometry data performed before cataract surgery;
- The actual measured refraction at the spectacle plane with the misaligned tIOL;
- The corneal front surface curvature;
- Optionally, the corneal back surface curvature;
- The power and measured axis of the tIOL.

Biometric data and tomographic data are normally acquired before cataract surgery, or before rotation-surgery. The corneal power may be derived either from keratometric data (corneal front surface curvature or keratometric data—thin cornea model) or alternatively from tomographic data (corneal front and back surface curvature or keratometric and back surface power—thick cornea model). Because the spherocylindrical refraction, corneal power, and tIOL are located in different planes, we require a vergence transformation strategy for spherocylindrical vergences. The principle of transforming spherocylindrical vergences through a homogeneous optical medium and to add up or subtract spherocylindrical vergences or refractive surfaces is described in detail in previous studies [3,4,15,16]. The calculation scheme used in this study was implemented in Matlab (Matlab version 2023a, MathWorks, Natick, MA, USA).

Input data

General data: Vertex distance (VD = 12 mm), keratometer index used for keratometry (nKm), refractive index of air (n = 1.0), refractive index of cornea (nC = 1.376), refractive index of aqueous humour (nA = 1.336), and refractive index of vitreous humour (nV = 1.336), as derived from the Liou–Brennan schematic model eye (LBME [23]). According to this LBME, we derived a keratometer index (nK = 1.329) such that the corneal power derived with a thick lens formula referenced to the corneal front vertex plane equals the keratometric power derived with a thin lens formula, as shown in a previous study [17,24].

Biometric data: Measurements of axial length (AL in mm), anterior chamber depth (ACD in mm, distance between the corneal front apex and the crystalline lens front apex), and the central thickness of the crystalline lens (LT in mm).

Refraction data: Measured refraction at the spectacle plane (sphere (REFSm in D), cylinder (REFCm in D), and cylinder axis (REFAm in degrees (°)) with the misaligned tIOL.

Corneal data: Corneal front surface curvature (radii in the flat meridian (R1a in mm at an axis RAa in degrees) and in the steep meridian (R2a in mm)) or keratometric power in the flat meridian (K1a in D at an axis KAa in degrees) and in the steep meridian (K2a in D); optionally corneal back surface curvature (radii in the flat meridian (R1p in mm at an axis RAp in degrees) and in the steep meridian (R2p in mm)) or back surface power in the flat meridian (P1p in D at an axis PAp in degrees) and in the steep meridian (P2p in D) together with central corneal thickness (CCT in mm). As an alternative to corneal front and back surface data from a tomographer, the corresponding total corneal power data (e.g., TCRP from Pentacam [Oculus, Wetzlar, Germany] or RealPower from Casia2 [Tomey, Nagoya, Japan]) could be entered as keratometric power data but without considering any additional correction for the back surface astigmatism.

IOL power: Power and measured axis of the tIOL (equivalent power (tIOLE in D), torus (tIOLT in D)) and measured axis (tIOLAm in degrees).

Data preprocessing

Effective lens position prediction: To avoid complexity we use a simple prediction model for the effective lens position (ELP in mm). Depending on the available biometric data, we could use the ACD and LT, the AL, or—if no biometry is available—the tIOLE together with the A constant of the implanted tIOL [18,19,20,21].

For the development of the ELP prediction model, we used a large clinical dataset containing 36,362 biometric measurements from the IOLMaster 700 (Carl-Zeiss Meditec, Jena, Germany), which have been uploaded in an anonymised fashion by several surgical centres between November 2017 and November 2025 to our IOLCon database (https://iolcon.org) for lens constant optimisation. After omitting incomplete records (e.g., records with missing Ra, Rp, CCT, ACD or LT), a total of *N* = 22,577 data were used to develop the ELP prediction models.

For the simplified ELP prediction model, we could use either a generalised sigmoidal model based on the AL measurement

$$ELP=f\left(A\right)+a+(b-a)\cdot (1+exp(-4\cdot log(3)\cdot (AL-c)/d)),$$

with left boundary a = 5.604, right boundary b = 5.184, inflexion point c = 23.32, and expansion factor d = 4.275, or a linear model based on the ACD and LT measurement

$$ELP=f\left(A\right)+ACD+0.38\cdot LT,$$

or a generalised sigmoidal model based on the equivalent power of the tIOL

$$ELP=f\left(A\right)+a+(b-a)\cdot (1+exp(-4\cdot log(3)\cdot (tIOLE-c)/d)),$$

with left boundary a = 5.157, right boundary b = 4.686, inflexion point c = 21.11, and expansion factor d = 10.880. For all of these 3 ELP prediction models we could use the same linear correction term for the A constant of the lens f(A) = −75.76 + 0.6368·A, where the A constant of the lens could be extracted, for example, from www.iolcon.org.

Corneal data: For the thin cornea model, if corneal data are provided in millimetres, the corneal power is calculated by K1a = 1000·(nK − 1)/R1a and K2a = 1000·(nK − 1)/R2a, and if corneal data are provided in dioptres, the corneal power is re-converted from nKm to nK by K1a = K1a·(nKm − 1)(nK − 1) and K2a = K2a·(nKm − 1)(nK − 1). For a thick cornea model, if corneal data are provided in millimetres, the corneal front surface power is calculated by P1a = 1000·(nC − 1)/R1a and P2a = 1000·(nC − 1)/R2a and the corneal back surface power by P1p = 1000·(nA − nC)/R1p and P2p = 1000·(nA − nC)/R2p, and if corneal data are provided in dioptres, the corneal front surface power is re-converted from nKm to nC by P1a = K1a·(nC − 1)/(nKm − 1) and P2a = K2a·(nC − 1)/(nKm − 1). For corneal back surface power, no conversion is required.

Optional consideration of surgically induced astigmatism (SIA) and statistical back surface astigmatism correction for the thin cornea model (CPA), both of which by convention do not affect the equivalent power of the cornea [3,15], were used. The SIA cylinder magnitude (SIAC in D) and axis of corneal incision (SIAA in degrees) are converted to the corresponding power values -SIAC/2 at axis SIAA and SIAC/2 perpendicularly. Accordingly, the CPA cylinder magnitude (CPAC in D) and axis (CPAA in degrees) are converted to the corresponding power values -CPAC/2 at axis CPAA and CPAC/2 perpendicularly.

In the following section we provide a step-by-step approach for our calculation based on transformation of spherocylindrical vergences.

**Figure 1:**
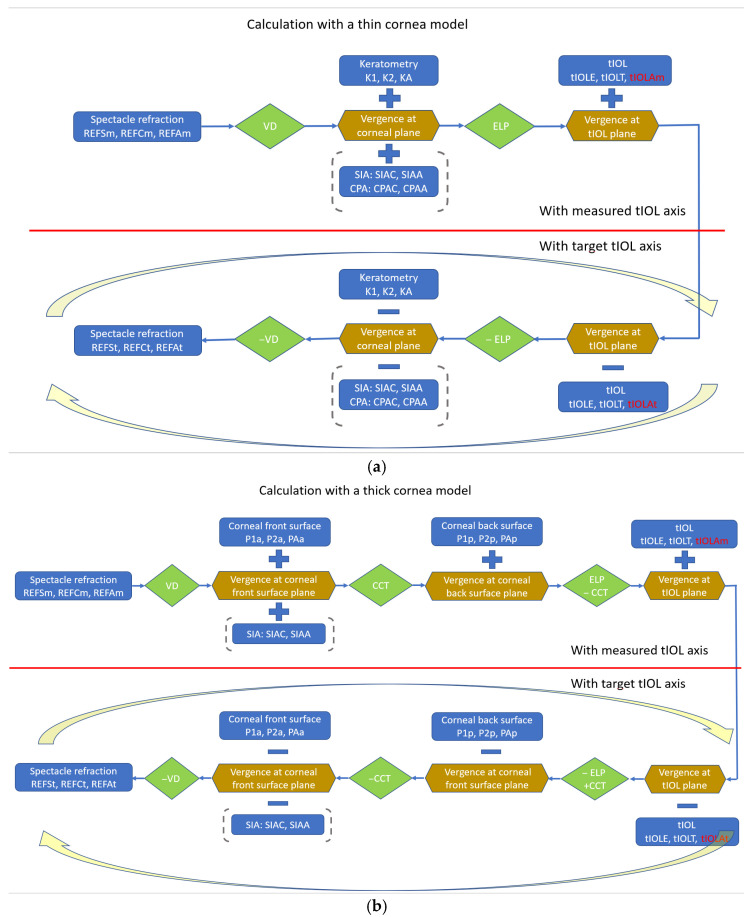
(**a**) Calculation scheme for the thin cornea model. The cornea is simplified to a thin lens characterised in terms of keratometric measures. In the forward calculation path (upper part of the graph), we extract the spherocylindrical vergence behind the lens plane with the measured toric axis, tIOLAm, and in the backward calculation path embedded in a nonlinear iterative optimisation loop (lower part of the graph), we derive the target lens orientation, tIOLAt, which yields the lowest refractive cylinder at the spectacle plane REFCt. (**b**) Calculation scheme for the thick cornea model. The cornea is represented with corneal front and back surface power measures. In the forward calculation path (upper part of the graph), we extract the spherocylindrical vergence behind the lens plane with the measured toric axis, tIOLAm, and in the backward calculation path embedded in a nonlinear iterative optimisation loop (lower part of the graph), we derive the target lens orientation, tIOLAt, which yields the lowest refractive cylinder at the spectacle plane REFCt.

Thin cornea model:

The calculation scheme based on transforming spherocylindrical vergences is shown in Figure 1a. In the forward calculation, based on the actual measurement of spectacle refraction, the vergence at the spectacle plane is transferred to corneal plane. At the corneal plane, we add up the corneal power and optionally SIA and/or CPA to consider changes in astigmatism due to cataract incision and corneal back surface astigmatism. The resulting vergence is transformed to the tIOL plane, where the tIOL power (with axis tIOLAm) is subtracted to obtain the spherocylindrical vergence behind the tIOL. Then, in the backward calculation, we add up the power of the rotated tIOL (with axis tIOLt), transfer back to the corneal plane, subtract the corneal power and optionally SIA and CPA, and transfer this vergence at the corneal plane back the to spectacle plane to obtain the spectacle correction. The backward calculation is organised in an optimisation loop which modifies tIOLAt to minimise the refractive cylinder REFC of the resulting spectacle refraction.

Thick cornea model

The calculation scheme for the thick cornea model is shown in Figure 1b. In the forward calculation, based on the actual measurement of spectacle refraction, the vergence at the spectacle plane is transferred to corneal front vertex plane. At the corneal plane, we add up the corneal front vertex power and transfer the vergence to the corneal back vertex plane. Corneal back surface power is added to the vergence at the corneal back surface, and the result is transferred to the tIOL plane, where we add the tIOL power (with axis tIOLAm) to obtain the spherocylindrical vergence behind the tIOL. Then, in the backward calculation, we subtract the power of the rotated tIOL (with axis tIOLt), transfer back to the corneal back surface plane, subtract the corneal back surface power, transfer this vergence to corneal front surface plane, and subtract the corneal front surface power. This vergence at the corneal front surface plane is again transferred to the spectacle plane to obtain the spectacle correction. The backward calculation is organised in an optimisation loop which modifies tIOLAt to minimise the refractive cylinder REFC of the resulting spectacle refraction.

Optimisation

The backward calculations were arranged in an optimisation loop, where the target tIOL axis tIOLAt was rotated to receive the lowest refractive cylinder value for the spectacle refraction. tIOLAt was initialised with the measured axis of the tIOL tIOLAm. The optimisation cycle was implemented using an iterative nonlinear sequential quadratic programming algorithm (SQP), as described in previous papers [24,25,26,27]. A step size tolerance of 1 × 10^−10^ and a function tolerance of 1 × 10^−12^ were used as the stopping criteria for the algorithm. In the final step we read out the target tIOL axis tIOLAt yielding the lowest refractive cylinder at the spectacle plane and the corresponding target refraction in terms of sphere (REFSt in D), cylinder (REFCt in D) and cylinder axis (REFAt in degrees). From the tIOLAt and tIOLAm, we calculate the amount of tIOL rotation (ΔtIOLA = tIOLAt − tIOLAm in degrees), and the potential relative and absolute gain in performance in terms of a relative reduction in the refractive cylinder (relative gain GainR: = 100·(1-REFCt/REFCm) in % and absolute gain GainA: = REFCm − REFCt).

## 3. Results

Here we would like to present the results of four clinical working examples: In the first example, we used keratometric power data to characterise a thin cornea model without consideration of SIA and CPA. For the ELP calculation, we used ACD and LT from the phakic eye. In the second example, we used keratometric curvature data to characterise a thin cornea model, but with consideration of SIA and CPA. For the ELP calculation, we used AL from the phakic eye. And in the last example, we used corneal front and back surface curvature data to characterise a thick cornea model. For the ELP calculation, we used the equivalent power of the implanted lens tIOLE. The input data for the four clinical working examples are listed in Table 1.

Example 1:

After re-conversion from nK0 to nK, the corneal power is calculated as 41.4138 D @ 15° and 43.8499 D @ 105°. From ACD, LT and the A constant, we calculate ELP = 5.1786 mm. The iterative optimisation gives us a rotation angle of 13.66 degrees (target axis tIOLAt = 98.86 degrees to minimise the refractive cylinder. The spectacle refraction is predicted to reduce from −0.5 D −1.0 D @ 50° to −0.9417 −0.1136 @ 98.66° after re-alignment of the tIOL axis to tIOLAt. The relative and absolute gain in performance GainR is 88.6% and GainA = 0.8864 D.

Example 2:

After correction with SIA and CPA, the corneal power is calculated as 40.0811 D @ 34.66° and 43.2984 D @ 124.66°. From AL and the A constant, we calculate ELP = 4.7154 mm. The iterative optimisation gives us a rotation angle of 8.34 degrees target axis tIOLAt = 28.34 degrees to minimise the refractive cylinder. The spectacle refraction is predicted to reduce from −2.0 D −1.5 D @ 11° to −2.0742 −1.3586 @ 28.34° after re-alignment of the tIOL axis to tIOLAt. The gain in performance is GainR = 9.4% and GainA = 0.1414 D.

Example 3:

The total corneal power referenced to the corneal front surface plane is 38.8715 D @ 84.83° and 44.1776 D @ 174.83°. For comparison, the keratometric power derived with the keratometer index nK yields 39.1517 D @ 85° and 43.8499 D @ 175°. From AL and the A constant, we calculate ELP = 4.8326 mm. The iterative optimisation gives us a rotation angle of 14.12 degrees (target axis tIOLAt = 154.12) degrees to minimise the refractive cylinder. The spectacle refraction is predicted to reduce from −1.0 D −2.5 D @ 95° to −1.936 −0.6289 @ 64.12° after re-alignment of the tIOL axis to tIOLAt. The gain in performance is GainR = 74.8% and GainA = 1.8711 D.

Example 4:

The total corneal power referenced to the corneal front surface plane is 41.5055 D @ 68.94° and 45.0614 D @ 158.94°. For comparison, the keratometric power derived with the keratometer index nK together with SIA yields 41.9577 D @ 69.16° and 45.2548 D @ 159.16°. From AL and the A constant, we calculate ELP = 4.6298 mm. The iterative optimisation gives us a rotation angle of −14.43 degrees (target axis tIOLAt = 160.57 degrees) to minimise the refractive cylinder. The spectacle refraction is predicted to reduce from 0.75 D −1.5 D @ 30° to 0.0807 −0.1503 @ 160.57° after re-alignment of the tIOL axis to tIOLAt. The gain in performance is GainR = 89.9% and GainA = 1.3497 D.

## 4. Discussion

Pre-existing corneal astigmatism can be permanently reduced or corrected with toric intraocular lenses during regular cataract surgery. In general, this correction option is better tolerated by the patient than spectacle or contact lens correction of corneal astigmatism. Other treatment options, such as laser vision correction or implantation of additional lenses into a phakic or pseudophakic eye, might be an alternative but require an additional surgical procedure.

However, tIOLs—even if they are properly aligned during surgery—show high amounts of residual astigmatism (>0.5 D) in over 1/3 of cases. Furthermore, they may show some rotation in the short or long term after cataract surgery, with the consequence that the degree of astigmatism correction is reduced. Even small rotation angles reduce the astigmatism correction significantly, and as a rule of thumb, if a tIOL with intended full astigmatism correction is rotated by 3/8/15/30 degrees away from its target axis, the astigmatism reduction is reduced to 90%/28%/52%/0%. tIOLs implanted in the capsular bag could typically be re-aligned between 4 weeks and 6 months after cataract surgery. However, the indication for a re-alignment depends on several environmental conditions, such as the condition of the capsular bag, corneal endothelium, or the lens optic and haptic design and material. After YAG capsulotomy, a re-alignment of the tIOL is quite difficult to impossible [10,11,12,13,14,28,29,30,31].

The refractive correction effect of a tIOL is not a constant for all eyes. Statements such as ‘a tIOL with a 1.5 D toricity corrects 1.04 D astigmatism at the corneal plane’ do not hold in general. The degree of astigmatism correction depends on the vergence at the corneal plane, the corneal power, and the ELP. In other words, we have to consider the refraction at the spectacle plane, the VD, corneal power and the ELP to determine the refractive correction effect of a tIOL. This translation ratio (e.g., 1.5/1.03 D = 1.4563) increases with the vergence at the corneal plane (from spectacle refraction and object vergence), corneal power and the ELP. In our working examples 1/2/3/4, this translation ratio derived from the cylindrical portion of the vergence at tIOL plane and (directly behind) corneal front vertex plane gives values of 1.422/1.345/1.359/1.384. We feel that the larger values that are typically provided by lens manufacturers (such as 1.4563 as mentioned for the Alcon SN6AT3 or SN6AT4; https://www.myalcon.com/de/professional/cataract-surgery/iols/acrysof-iq-monofocal-and-toric/, accessed on 3 January 2026) are derived with classical formulae, where the overestimation of corneal power (from a too high keratometer index [17,24]) has to be compensated for by an adjustment of the ELP to posteriorly generate proper IOL power values [19,20,21].

In the present study we used paraxial optics and a simple vergence transformation [3,4,16] to develop a prediction strategy for the simulation of a tIOL adjustment. Based on the measured spherocylindrical refraction at the spectacle plane, the measured corneal curvature or power, the labelled equivalent and toric power of the tIOL and the measured tIOL axis, together with simplistic ELP models, we developed a concept to extract the amount of axis rotation of the tIOL which minimises the refractive cylinder at the spectacle plane after re-alignment. To make our calculation concept flexible for clinicians, we showed how to consider keratometric measurement data (keratometric curvature data in mm or power data in D) to characterise a thin cornea model or tomographic corneal front and back surface data (CCT together with corneal front and back surface curvature data in mm or keratometric and back surface power data in D) [3,4,15], and three alternatives for ELP prediction models based either on ACD and LT, on AL, or on tIOLE where biometric data are unavailable.

The step-by-step approach is shown in a block diagram in Figure 1. In the forward calculation path, we extract the vergence directly behind the tIOL using the measured tIOL axis. And in the backward calculation path, we optimise the tIOL axis to minimise the resulting refractive cylinder at the spectacle plane. In principle, this concept could consider statistical vector corrections such as SIA (both for the thin and thick cornea model) or CPA (only for the thin cornea model), but in reality, the impact of SIA and CPA on the result is quite limited. With the predicted amount of axis rotation or the target axis of the tIOL, we are able finally to predict the spherocylindrical refraction at the spectacle plane and the gain in performance in terms of reduction in refractive cylinder due to the re-alignment of the tIOL axis. These data could assist the clinician with the indication for the re-alignment procedure.

The ELP prediction models have been derived from a large clinical dataset based on the ELP prediction with the Castrop formula [3,18,22]. Alternatively—depending on the available clinical data—the user could consider the phakic ACD and LT data, the AL data, or the tIOLE data. ACD, LT and AL data require preoperative biometric measures, whereas the equivalent power of the tIOL is available anyway and is required for our calculation scheme. The ELP prediction based on AL or tIOLE uses a sigmoidal function to restrict the dynamic range for the ELP. However, the user could easily replace our ELP prediction models with any preferred custom ELP prediction.

For our calculation concept, we used an internal keratometer index of nK = 1.329. This keratometer index has been extracted from the cornea of the Liou–Brennan schematic model eye [23] by calculating the corneal power referenced to the corneal front apex plane [17,24]. As a consequence, if keratometric power data are used which have been derived with any other keratometer index (e.g., nK0 = 1.332 or nK0 = 1.3375), data entries with keratometric power have to be converted to nK [15]. Nevertheless, in general the calculation scheme could be customised to any internal keratometer index nK if necessary.

To show the applicability of our calculation concept, we presented four clinical working examples: In example 1 we used keratometric power data without any additional correction with a SIA and CPA to define a thin cornea model. In example 2 we used keratometric curvature data and considered SIA and CPA to define a thin cornea model. In example 3 we used curvature data from both surfaces and corneal thickness to define a thick cornea model, and in example 4 we used keratometric power data from the corneal front surface and corneal back surface power data together with a vectorial SIA correction to define a thick cornea model.

The results of our examples clearly show that in examples 1, 3 and 4, there is a clear indication for re-alignment of the tIOL. In example 1 the refractive cylinder at the spectacle plane is expected to be reduced by 89% from 1.0 D to 0.11 D, in example 3 by 75% from 2.5 D 0.63 D, and in example 4 by 90% from 1.5 D to 0.15 D. In contrast, in example 2 the gain in performance is rather low at 9%, and the spectacle cylinder is only expected to be reduced from 1.5 D to 1.36 D. Therefore, a re-alignment of the tIOL might not be indicated. However, in addition to the expected gain in performance after tIOL re-alignment, we also obtain information on the axis of the refractive cylinder after adjustment. This might also be helpful for the clinicians for indication making, as in general, axis orientations with-the-rule or against-the-rule might be preferred over oblique axes of the refractive cylinder [15,16].

In clinical practice, there are three WEB-based planning tools for re-alignment of toric capsular bag lenses: For https://www.astigmatismfix.com/ (accessed on 3 January 2026), developed by Li Wang and Douglas Koch, and https://www.toricaligner.com/standard (accessed on 3 January 2026), developed by Daten Buonsante, the user has to enter corneal power together with the astigmatism correction effect of the tIOL converted to corneal plane. As this conversion from lens plane to corneal plane depends on the entrance vergence at the corneal plane, together with the corneal power and the ELP, this concept might require additional calculations to produce the proper conversions. Recently, Damien Gatinel developed a planning tool, https://www.gatinel.com/essai-2-2/ (accessed on 3 January 2026), which uses the entrance vergence at the corneal plane together with the corneal power and the measured axial position of the tIOL in the eye. However, the calculation strategy behind these planning tools has not been disclosed, which makes a comparison with our calculation strategy difficult.

However, our study has some limitations: First, we used simple paraxial vergence calculations: where tomographic datasets are available, full aperture raytracing concepts that make use of lens decentration and tilt might be more powerful and additionally could give some insight into the image performance with the measured as well as target tIOL axis as a function of pupil size. Second, we used relatively simplistic ELP prediction models based either on the location of the crystalline front and back vertex in the phakic eye, axial length, or on the equivalent power of the implanted lens. There might be more advanced and powerful ELP prediction models which could enhance the performance of the calculation concept. Third, we used a keratometer index internally, which does not match the most commonly used Javal or Zeiss keratometer index. However, we feel that our lower keratometer index tends to less overestimation of corneal power, and if necessary the keratometer index could be easily customised in the user implementation of this calculation concept. Fourth, case numbers are needed to show if additional information on cortical perception can be gained from the subjective refraction. Fifth, this study was concerned specifically with correction of tIOL axis misalignment, but this is of course only one of a number of factors affecting the quality of the final corrected vision. Other factors such as spherical equivalent error, axis stability, and higher-order aberrations (e.g., contrast sensitivity, anamorphic distortion) are also relevant, and a full consideration of all such factors should naturally form part of any clinical decision-making process. And finally, the main focus of this study was to develop a calculation strategy which could be implemented in any consumer software or online calculator rather than showing clinical results. A clinical validation is mandatory before broad clinical use.

## 5. Conclusions

In conclusion, in this paper we present a calculation concept to predict the best axis of a toric intraocular lens to minimise the residual refractive cylinder at the spectacle plane. This concept is based on the actual refraction, the actual keratometry or keratometry before cataract surgery, the labelled equivalent and toric power of the lens, and the measured axis of the lens. The concept could deal with corneal curvature or power data for the front surface, or for the front and back surface together with corneal thickness, and predicts the spectacle refraction in terms of sphere, cylinder and cylinder axis, together with the potential gain in performance in terms of refractive cylinder reduction. A clinical evaluation of this calculation concept in a prospective study will be required to show the applicability in a clinical setup.

## Figures and Tables

**Table 1:** Input parameters for the 4 clinical working examples. Example 1 uses standard keratometry in dioptres (flat meridian K1 at axis Ka, steep meridian K2) and anterior chamber depth (ACD) and lens thickness (LT) for prediction of the axial lens position. Example 2 uses standard keratometry in millimetres (flat meridian R1a at axis Raa, steep meridian R2a) and axial length for prediction of axial lens position. Example 3 uses corneal front and back surface data (R1a, R2a, RAa and back surface flat radius R1p at axis RAp and steep radius R2p) and equivalent lens power tIOLE for prediction of axial lens position. Example 4 uses keratometric and corneal and back surface data (K1a, K2a, KAa and back surface flat power P1p at axis PAp and steep radius P2p) and AL for prediction of axial lens position. With keratometry, statistical corneal back surface astigmatism correction (magnitude CPAC at axis CPAA) could be considered. Surgically induced astigmatism (magnitude SIAC at incision axis SIAA), measured refraction at the spectacle plane (sphere REFSm and cylinder REFCm at axis REFAm), and implanted toric lens (equivalent power tIOLE and torus tIOLC at measured axis tIOLAm) are examined.

	Input Parameter	Example 1	Example 2	Example 3	Example 4
Biometry	Axial length AL in mm		25.80		22.15
	Anterior chamber depth ACD in mm (phakic)	3.15			
	Lens thickness LT in mm (phakic)	4.45			
Corneal data	K1 in D @ KA in degrees	42.5 @ 15			43.0 @ 70
	K2 in D	45.5			46.5
	nKm	1.3375			1.332
	P1p in D @ PAp in degrees				−6.6 @ 80
	P2p in D				−7.0
	R1a in mm @ RAa in degrees		8.2 @ 35	8.4 @ 85	
	R2a in mm		7.6	7.5	
	R1p in mm @ RAp in degrees			6.6 @ 95	
	R2p in mm			6.5	
	SIAC in D @ SIAA in degrees	-	0.2 @ 85	-	0.15 @ 0
	CPAC in D @ CPAA in degrees	-	0.25 @ 0	-	-
	Corneal thickness CCT in mm			0.52	0.55
	Cornea model	Thin cornea	Thin cornea	Thick cornea	Thick cornea
Measured refraction	REFSm in D	−0.5	−2.0	−1.0	0.75
	REFCm in D	−1.0	−1.5	−2.5	−1.5
	REFAm in degrees	50	11	95	30
Toric IOL with measured axis tIOLAm	A constant	119.5	118.3	118.6	118.8
	tIOLE in D	21.5	14.5	17.5	24.5
	tIOLC in D	3.0	3.75	6.0	4.25
	tIOLAm in degrees	3	20	140	175

## Data Availability

No new data were created for this study, and the input parameters to retrieve the results for the clinical examples are mentioned in the text.
